# On Differential Mechanisms for Underactuated, Lightweight, Adaptive Prosthetic Hands

**DOI:** 10.3389/fnbot.2021.702031

**Published:** 2021-10-18

**Authors:** Geng Gao, Mojtaba Shahmohammadi, Lucas Gerez, George Kontoudis, Minas Liarokapis

**Affiliations:** ^1^New Dexterity Research Group, Department of Mechanical Engineering, University of Auckland, Auckland, New Zealand; ^2^The Bradley Department of Electrical and Computer Engineering, Virginia Tech, Blacksburg, VA, United States

**Keywords:** upper-limb prosthesis, differential mechanisms, robot hands, grasping, underactuated mechanisms

## Abstract

Over the last decade underactuated, adaptive robot grippers and hands have received an increased interest from the robotics research community. This class of robotic end-effectors can be used in many different fields and scenarios with a very promising application being the development of prosthetic devices. Their suitability for the development of such devices is attributed to the utilization of underactuation that provides increased functionality and dexterity with reduced weight, cost, and control complexity. The most critical components of underactuated, adaptive hands that allow them to perform a broad set of grasp poses are appropriate differential mechanisms that facilitate the actuation of multiple degrees of freedom using a single motor. In this work, we focus on the design, analysis, and experimental validation of a four output geared differential, a series elastic differential, and a whiffletree differential that can incorporate a series of manual and automated locking mechanisms. The locking mechanisms have been developed so as to enhance the control of the differential outputs, allowing for efficient grasp selection with a minimal set of actuators. The differential mechanisms are applied to prosthetic hands, comparing them and describing the benefits and the disadvantages of each.

## 1. Introduction

The human hand is a powerful tool enabling humans to perform a wide range of tasks that range from interacting with objects used in daily living to executing gestures in social activities. According to Ziegler-Graham et al. (2008), approximately 540,000 amputees have suffered from upper limb loss in the US, with the expected projections to be doubled by 2050. In Italy and the UK, approximately 3,500 and 5,200 upper limb amputations occur every year (Cordella et al., 2016). Amputations can have a detrimental effect on an amputee's quality of life, preventing them from executing critical grasps needed in activities of daily living (ADL).

The latest technological advancements have helped improve prosthetic hand development toward becoming increasingly dexterous devices. Despite this, design tradeoffs between the dexterity of the prosthesis and weight, form factor, and cost of the device still exist (Bicchi, 2000). Although there are highly dexterous robot hands capable of emulating the dexterity of the human hand (Kochan, 2005; Grebenstein et al., 2010; Cerulo et al., 2017), the number of independent degrees of freedom (DOF) and the actuators utilized make it challenging to control such devices without compromising the weight, form factor, and affordability needed by amputees so as to adopt these hands for ADL.

In order to develop affordable, lightweight, and compact prostheses, researchers have employed synergistic methods (Xiong et al., 2016; Della Santina et al., 2018) and adaptive systems through the use of differential mechanisms that reduce the number of actuators needed to control multiple fingers simultaneously. Differential mechanisms in adaptive robotic and prosthetic hands distribute a single input torque to numerous fingers, resulting in stable and efficient grasps (Birglen et al., 2007). Such mechanisms allow the fingers to passively adapt to object shapes during the grasp, maximizing the number of contact points. The maximization of the contact areas during grasping also leads to the maximization of the grasping stability (Liarokapis et al., 2015). An even force/torque transmission in prosthetic and robotic devices can be achieved by different types of mechanisms, such as geared differentials, ball differentials, combination of pulleys, whiffletree mechanisms, and fluidic differentials. The geared differential is the most popular mechanism for force/torque transmission, and it is applied in different fields. This system can be implemented with different gears, such as planetary gears, spur gears, and bevel gears. The geared differential's main advantages is the ability to handle large torques and constant torque output regardless of the configuration. However, the added complexity of gears can make the implementations large and heavy (Martin et al., 2004; Birglen et al., 2007). Different from the geared differentials, ball differentials can be easily miniaturized by replacing a set of gears with several miniature ball bearings rotating between two plates (Keller et al., 2015). On the other hand, ball differentials require constant maintenance and can handle less torque than the traditional gear differentials.

Another type of differentials, the pulley differentials, use multiple moving pulleys to convert a single input into multiple outputs. Selection of pulley diameters and arrangements can be made to offer a mechanical advantage to the system so as to improve the force exertion capabilities (Ma et al., 2013). However, the main disadvantage of the floating pulley systems is that they need to maintain tension in the cables as loose cables can cause them to escape from the pulleys compromising the tendon routing. Similar to the pulley differential, in the whiffletree differential, a series of cables/tendons are used to suspend a floating mechanism/bar, which distributes a force equally across the outputs. Instead of pulleys, the whiffletree differential uses levers/bars. The tendons are attached to the end of the levers. Although the design is compact, the levers can limit the range of motion that is achievable by the differential.

Finally, an unusual type of differential mechanism applied to robotic devices is the fluidic t-pipe differential. This differential utilizes fluids such as air, water, or oil to transmit force from an input to multiple outputs through t-pipes (Birglen and Gosselin, 2006). Unlike traditional differentials, the ability of the fluid used to compress can provide actuation compliance to the system. Although such a differential mechanism allows for the absorption of shocks depending on the selected type of fluid, leaking phenomena typically affect the performance and robustness of the mechanism making it hard to repair and maintain and leaks may damage neighboring components.

Many authors have employed differential mechanisms in prosthetic hands. In Kontoudis et al. (2015) and Leddy and Dollar (2018), the authors introduce robotic hands that use whiffletree differential mechanisms to control the robot fingers using a single motor. The whiffletree differentials evenly transmit the forces among the fingers. However, they require additional space to operate, and a precise tendon tension calibration is needed. In Gosselin et al. (2008) and Belter and Dollar (2013), the authors describe the design of robot hands that uses pulley differentials and one actuator to actuate five fingers simultaneously. Multiple objects can be grasped with these lightweight designs. Similarly, the pulley mechanisms take a considerable amount of space in the robotic hands. Additionally, the friction between the tendons and the pulleys reduces the efficiency of the system.

In Xu et al. (2015), the authors proposed a continuum differential mechanism applied to a prosthetic hand. The particular robotic device employs one actuator and combines a rack-pinion-based system and the traditional whiffletree mechanism to drive five fingers. In Cheon et al. (2014), the authors proposed a robotic hand using a differential gear mechanism to distribute one input from the actuator to the finger joints. In Cipriani et al. (2011), Mitsui et al. (2013), and Chen et al. (2015), the authors used elastic elements connected in-between the driveshaft and the actuated fingers to achieve an adaptive transmission, which allows the robot hand to conform to the grasped object. Although the transmission facilitates adaptive grasping, the system requires additional force to be applied to deform the elastic element and produce adaptive behaviors at the outputs, consuming more energy compared to other differential systems. One of the advantages of the aforementioned robotic grippers is that they can grasp a wide range of objects without requiring complex control algorithms or force sensors at the finger pads. Additionally, the use of a minimal number of actuators, such a design advantage makes this category of robot hands intuitive to operate and highly affordable.

In order to expand the capabilities of underactuated devices, appropriate locking mechanisms have been employed by researchers to facilitate the execution of various grasp poses and gestures in prosthetic hands. In Belter and Dollar (2013), the authors proposed the use of a bistable ratchet locking mechanism to enable control over the opposition of the thumb allowing for four independent grasping postures to be achieved with a single actuator. In Baril et al. (2013), the authors designed mechanical selectors, which are capable of obstructing the motion of a whiffletree differential allowing for three grasping modes to be executed with a single actuator by adjusting a slider-selector with the intact hand. However, this design is limited to a maximum of three grasping postures requiring the user to switch between different slider-selectors to achieve alternative grasping postures. In Chu et al. (2008) on the other hand, the authors used a cam ball clutch lock the robotic fingers in various configurations to conserve motor power. However, when wedging the balls into the cam to prevent further motion, a high wear rate from the friction is experienced in the mechanism. This wearing effect limits the materials that can be used in prosthetic hands compromising their durability, which is of critical importance.

In this paper, we present two different types of differential mechanisms and various manual and automated selectively lockable differential mechanisms that can be applied to underactuated, lightweight, adaptive prosthetic hands. The proposed designs are experimentally evaluated, and we also compare them, discussing the benefits, applicability, and disadvantages of each of them. The rest of the paper is organized as follows: section Design presents the designs of the four types of differential mechanisms, section Experiments and Results details the experimental setup used for the tests and presents the experimental results, section Discussion discusses the advantages and disadvantages of the differentials developed, while section conclusion concludes the paper.

## 2. Design

In this section, we present the designs of the proposed differential mechanisms as well as the designs of the manual and automated lockable mechanisms that have been implemented and integrated into the developed differentials to provide control over the outputs.

### 2.1. Four-Output Gear Differential

**Figure 1:**
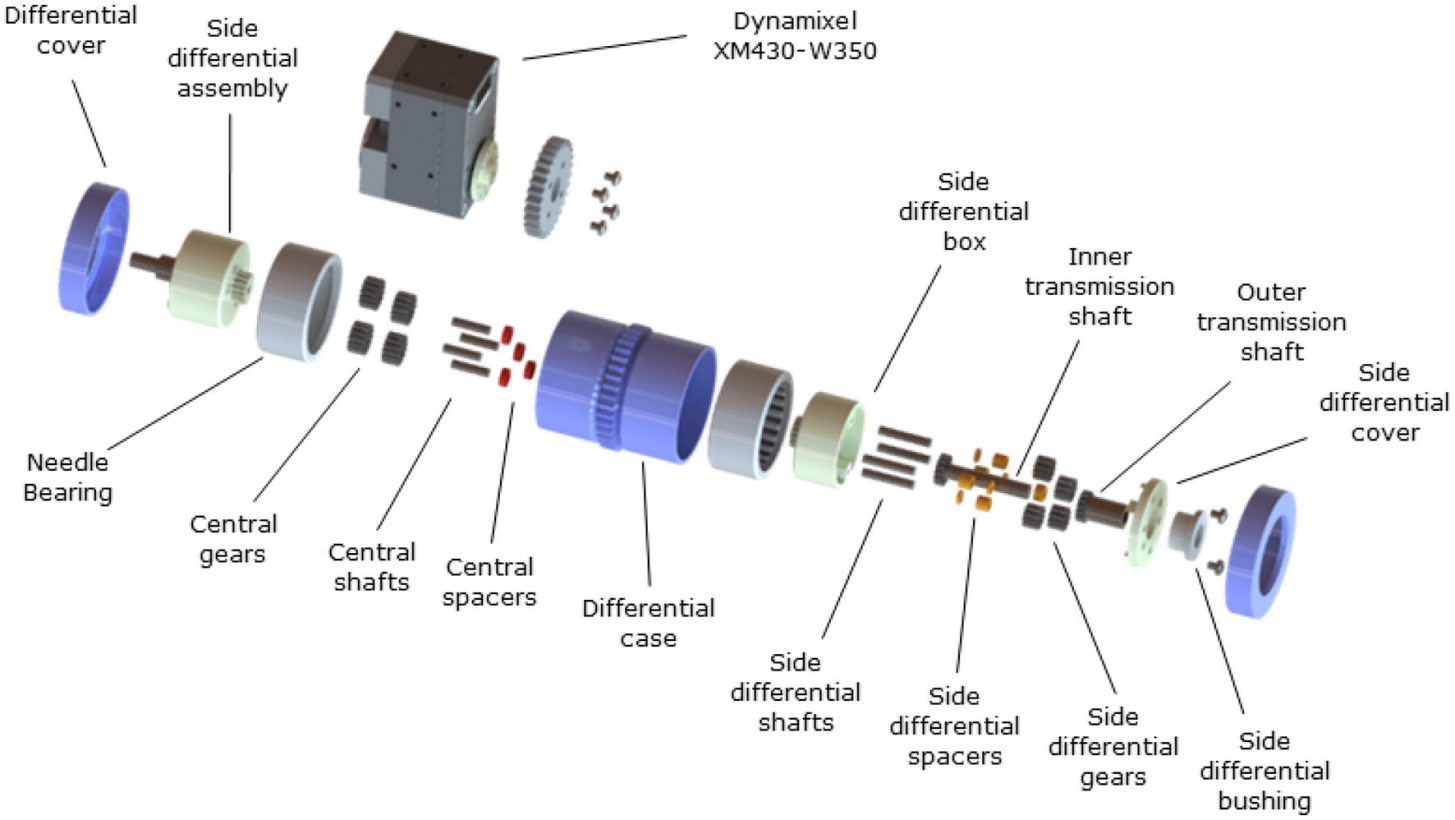
The four-output gear differential consists of three main structures: a central barrel and two lateral assemblies. The central barrel is composed of the differential case, two needle bearings, and four central gears, shafts, and spacers. The integrated gear on the differential case allows input torque from the Dynamixel XM430-W350-R motor to provide power to the mechanism. Each lateral assembly is comprised of an inner and outer transmission shaft, where the outer shaft is hollow to facilitate the inner shaft. Bushings and bearings in the system allow the system to rotate with minimal friction and in an efficient manner.

**Figure 2:**
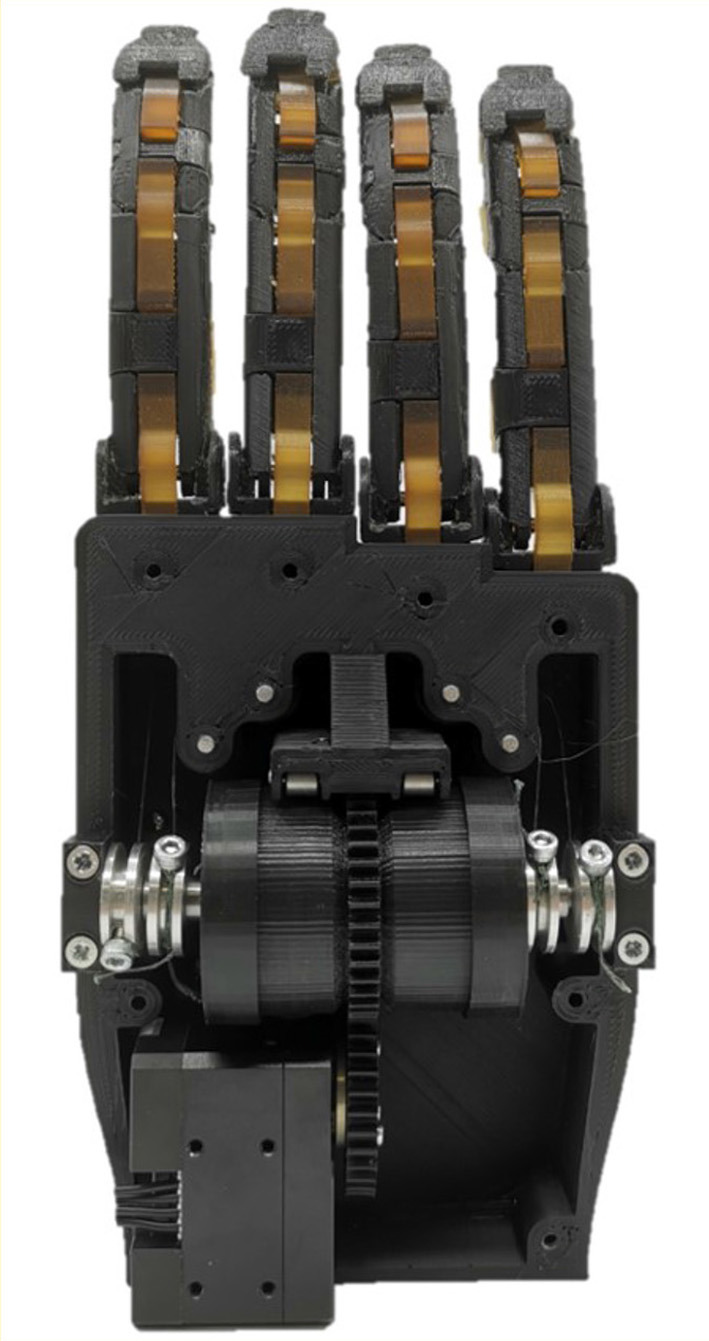
The four-output gear differential can be integrated into a prosthetic hand with the four outputs connected to the index, middle, ring, and pinky fingers. A single motor is used to distribute the load of the actuator to the four fingers through the outer gear module of the differential case.

**Figure 3:**
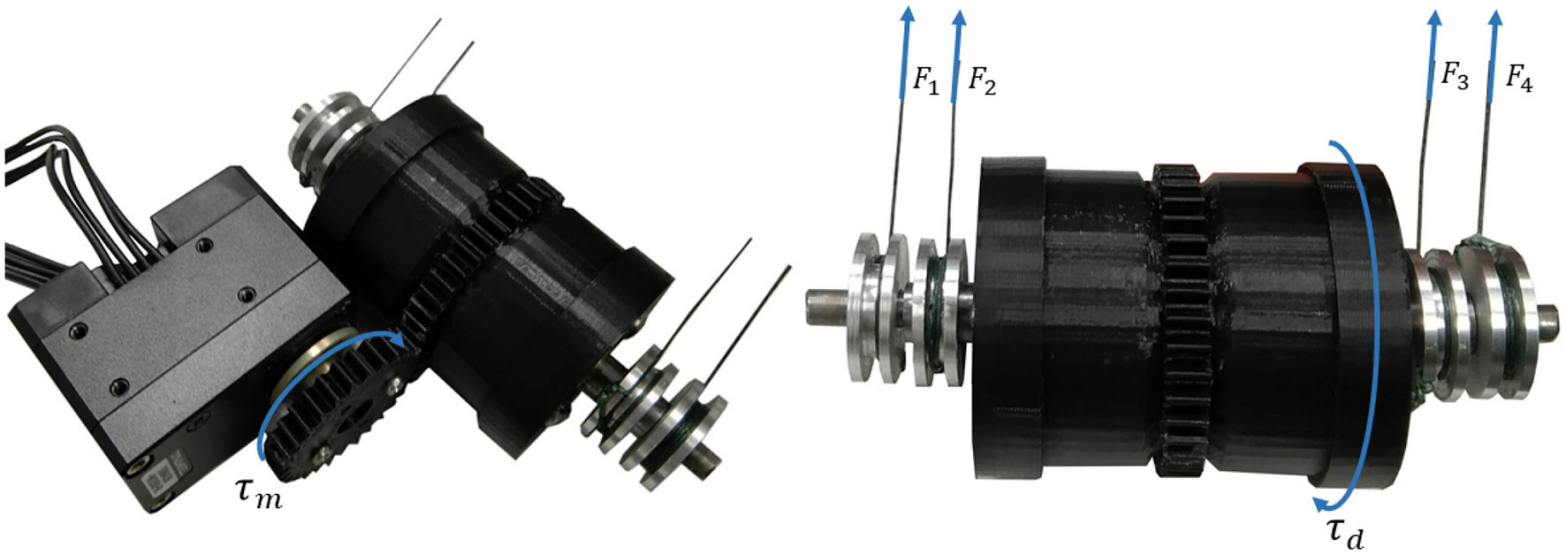
The four-output geared differential is driven by an input torque provided by the motor (τ_*m*_), which drives the differential generating a torque τ_*d*_. The torque τ_*d*_ is then evenly distributed across the four outputs of the differential: *F*_1_, *F*_2_, *F*_3_, and *F*_4_.

The four-output gear differential mechanism is composed of three main parts, a central barrel, and two different lateral assemblies, as shown in Figure 1. The central barrel is composed of a plastic cylinder, two needle bearings, and a combination of four spur gears that operate as a spur gear differential (Biermann et al., 2013). The outside geared ring is used to provide the torque input. Each lateral assembly is composed of six gears, two steel shafts, and a plastic case with a geared tip connected to the central barrel. A spur gear is connected to the end of the inner and outer shaft. Each lateral assembly has two shafts directed to the same side, an inner and an outer shaft. The outer shaft has a hollowed center where the inner shaft is placed, allowing both shafts to rotate with minimal friction. The bearings in the central barrel allow for the free rotation of the lateral assemblies. Such a design choice guarantees that all four shafts are placed on the same axis that facilitates a four-output gear differential operation. Figure 2 shows the position of the differential when incorporated into a prosthetic hand. The operation of the four-output gear differential is depicted in Figure 3.

In order to determine the most suitable motor and the gear ratio required between the motor and the differential mechanism, the maximum applicable forces of each output were calculated by applying (Equations 1–5). More precisely, τ_*d*_ is defined as the torque applied to the differential that is divided into four outputs, τ_1_, τ_2_, τ_3_, and τ_4_ (Equation 1). The torque is equally distributed among the outputs, as shown in Equation (2).

(1) $$\begin{array}{l} \tau_{d}=\tau_{1}+\tau_{2}+\tau_{3}+\tau_{4} \end{array}$$

(2) $$\begin{array}{l} \tau_{1}=\tau_{2}=\tau_{3}=\tau_{4}=\frac{\tau_{d}}{4} \end{array}$$

The output torque can be written in terms of the tendon tension (tangential force) and the radius of the pulley (Equation 3). The radius of all pulleys are the same, so the tension of all tendons will also be the same.

(3) $$\begin{array}{l} \tau_{i}=F_{i}r_{i} \end{array}$$

The torque of the differential is proportional to the torque applied by the motor, τ_*m*_, being multiplied by the gear ratio, *i*_*g*_, between the motor gear and the differential, as follows:

(4) $$\begin{array}{l} \tau_{d}=i_{g}\tau_{m} \end{array}$$

The force transmitted to each tendon can be written as shown in Equation (5).

In the differential proposed, the motor can apply a torque up to 3 N.m, the pulley channel has a diameter of 14 mm, and the gear ratio is 1.26. Thus, a maximum force of about 135 N can be achieved by each tendon, as follows:

(5) $$\begin{array}{l} F_{i}=\frac{i_{g}\tau_{m}}{4r_{i}}. \end{array}$$

### 2.2. Series Elastic Differential

**Figure 4:**
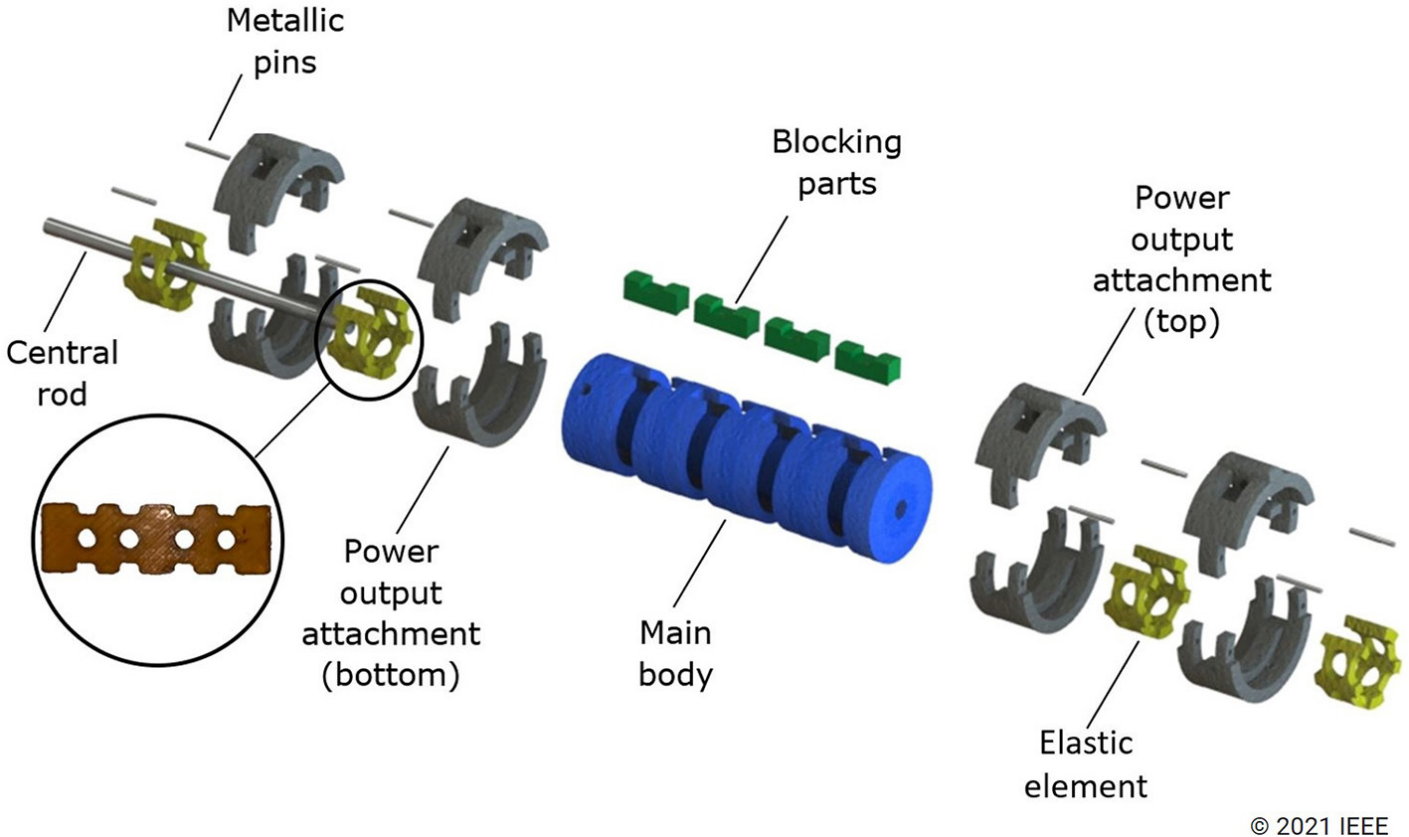
Exploded view of the proposed differential mechanism and robotic gripper. Elastic elements (yellow parts) inserted in slots of the main body. Then, power output attachments are inserted in the same slots over the elastic elements. Finally, plastic pieces (green parts) are inserted in the same slot to block the rotation of the elastic elements. The metallic rod at the center of the main body is used to prevent bending under heavy loads.

**Figure 5:**
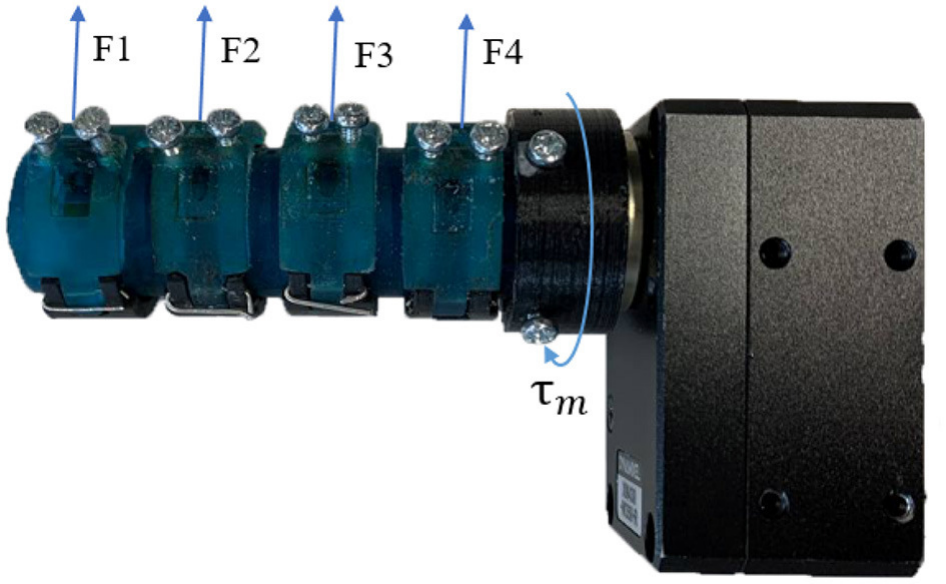
The four-output series elastic differential is driven by an input torque provided by the motor (τ_*m*_). This torque is evenly distributed across the four series-elastic elements that allow the gripper to conform to the object shape by getting proportionally compressed.

The series elastic differential extends the work presented in Shahmohammadi and Liarokapis (2021) and is composed of a rod-shaped main bar with four round slots in it. Elastic elements (made out of urethane rubber Smooth-On PMC-780) are placed inside each slot and then a rotating attachment is inserted inside the slots behind the elastic elements. Finally, the slots are blocked by a plastic piece to make sure that the elastic elements cannot rotate freely. Figure 4 shows an exploded view of this differential. This differential distributes the torque from the single motor (τ_*m*_) to the four series-elastic outputs (see Figure 5). Depending on the compression of the elastic element and the element properties, the output forces of the differential mechanism can vary significantly. The developed differential consists of four outputs that can be connected to the index, middle, ring, and pinky fingers of an anthropomorphic hand with the thumb being controlled separately.

This differential can work in two different modes: “Compliance Mode” and “Power Mode.” The Compliance Mode transitions through three stages during grasping. Initially, the force at the output is lower than the required force to compress the elastic element (fingers move in sync with each other). The second stage starts upon contact with the object's surface. At this stage, the acting forces on the elastic element become higher, and eventually, they start compressing it, offering the required grasping adaptability between the fingers. During this compression stage, the output attachment does not move since the required force for compressing the elastic element is lower than the acting force on the finger. This allows the remaining non-contacting outputs to continue moving. The elastic material keeps compressing until the required force is again higher than the force acting on the finger. When all fingers have made contact with the object's surface, forcing all elastic elements to reach their maximum compression, the outputs will start to move at the same speed again. In Power Mode, the main body rotates away from the elastic element (counterclockwise) and directly establishes contact with the hard stop end of the output attachment. By doing this, there are no energy losses due to contact with the elastic element, and the exerted forces are higher at the output. This mode is suitable for situations when compliance is not necessary.

**Figure 6:**
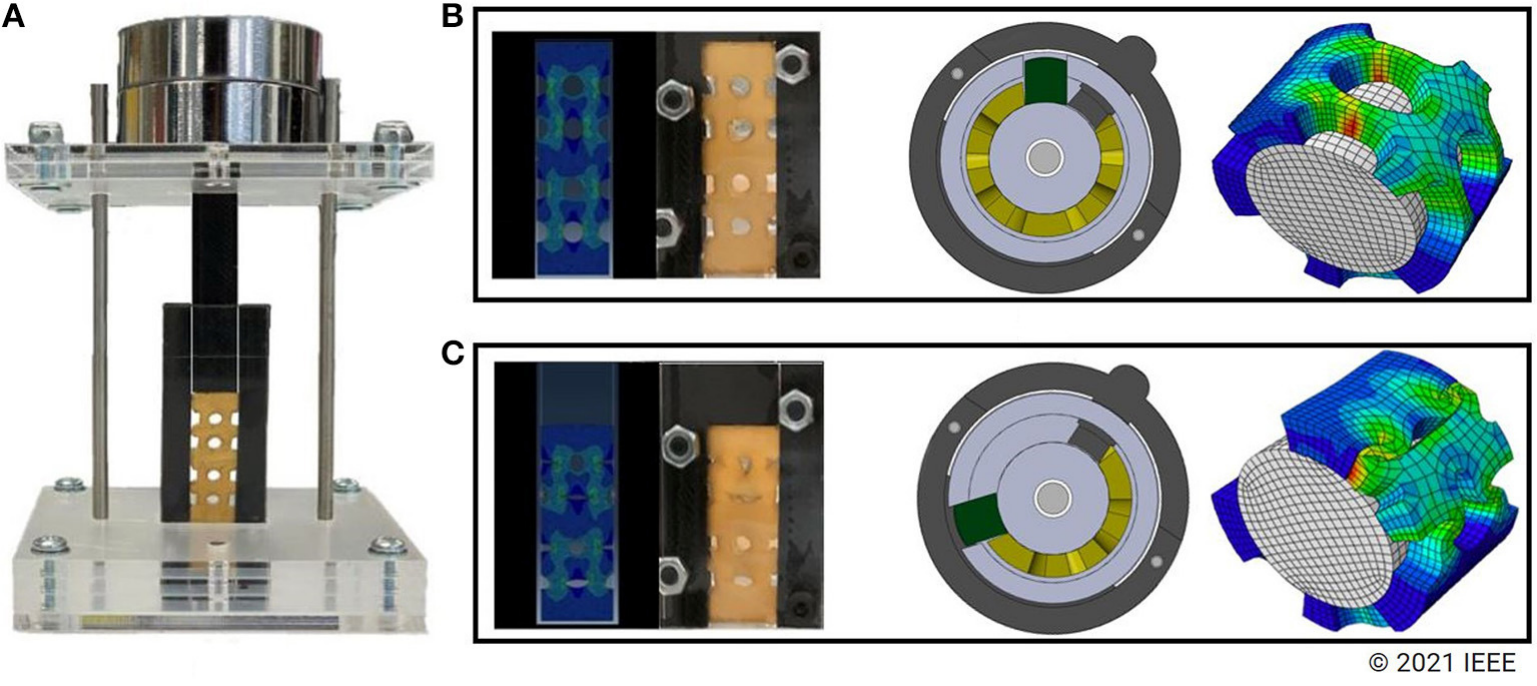
A uniaxial compression test was used to compare the simulated and the real compression of the elastic elements. **(A)** Presents the experimental setup, while **(B,C)** present the elastic element before and after compression respectively (both in simulation and reality).

To evaluate the elastic elements' behavior during loading, finite element modeling (FEM) was used to simulate the compression behavior. For the FEM analysis, the Abaqus simulation software was used with Mooney-Rivlin equations for hyperelasticity. This simulation allowed the calculation of how much force is required for initiating the compression of the elastic elements. More precisely, for an elastic element with 1.8 mm thickness, the compression starts at 3 N of force, which is small enough for a delicate grasp yet large enough to facilitate the successful execution of various grasps. Then we experimentally validated the accuracy of this number by performing a uniaxial compression test. The needed force can be easily adjusted by changing the thickness of the elastic element. Figure 6 presents both the simulation and the experiment conducted for comparison purposes.

### 2.3. Selectively Lockable Differentials

The design of the selectively lockable differential is motivated by the multiple grasping strategies that the human can choose for a given task. For that reason, we have proposed a mechanism based on the well-known whiffletree differential and the two new differentials that we have proposed. The use of a locking mechanism allows the user to select a grasp strategy from a wide range of possible combinations (Kontoudis et al., 2015).

#### 2.3.1. Manual Selectively Lockable Differentials

The manual selectively lockable differential mechanisms can block the motion of each finger, using a simple locking mechanism that works like a button, allowing the user to select in an intuitive manner the desired finger combinations and implement different grasping postures or gestures. When the buttons are pressed they elongate and obstruct the motion of the differentials.

**Figure 7:**
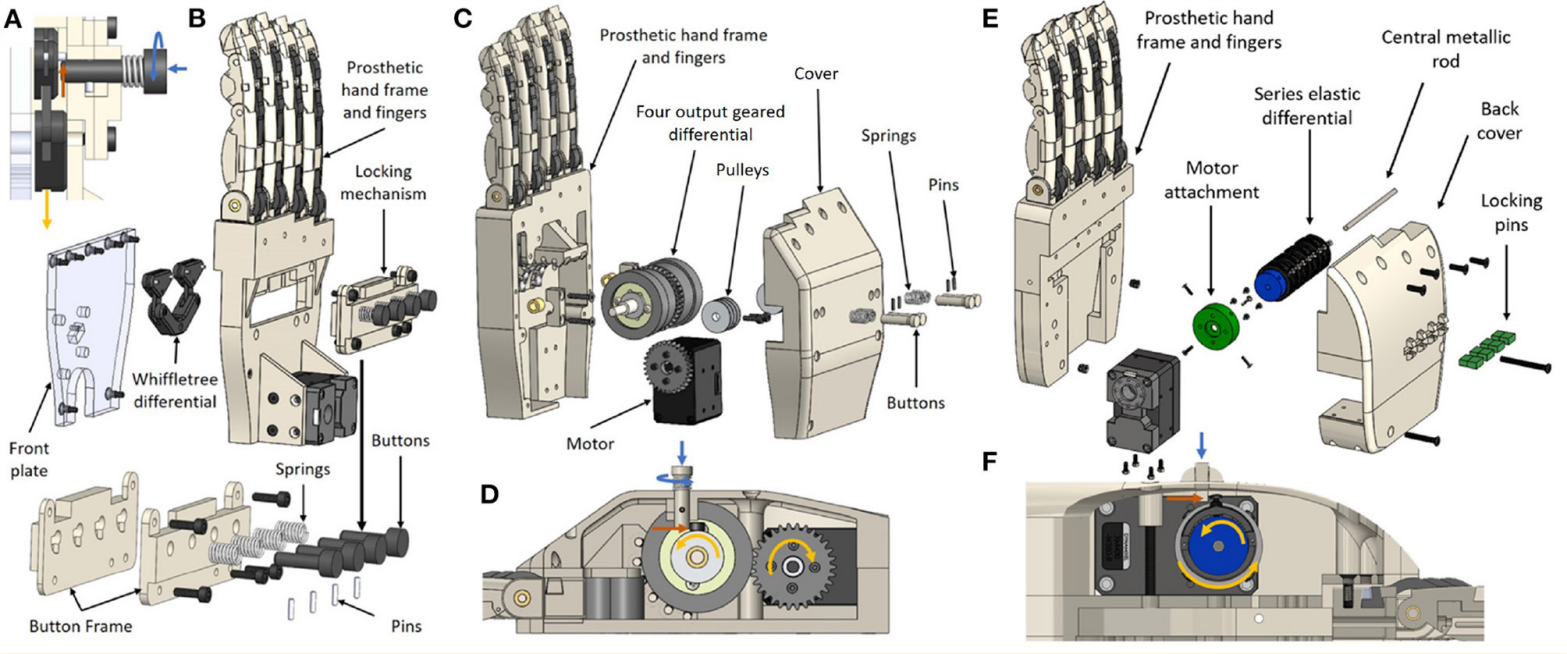
The manual selectively lockable mechanism has been integrated into three differentials: the whiffletree differential **(A,B)**, the four output geared differential **(C,D)**, and the series elastic differential **(E,F)**. **(A)** Illustrates how the locking mechanism blocks a selected output on the whiffletree differential. Button locking is executed through a pushing and twisting action, which engages the button for locking (this is shown with the blue arrows). When the whiffletree is actuated (represented by the yellow arrow), the button provides a blocking force (orange arrow) holding the selected output in place. **(B)** presents how the manual locking mechanism and the whiffletree differential are integrated into the prosthetic hand. Similarly, **(C)** presents the structure of the prosthetic hand when the lockable four output geared differential is used. The locking mechanism utilizes a similar button mechanism to block the motion of a pulley in the four output geared differential, as depicted in **(D)**. The exploded view of the series elastic differential is presented in **(E)**, showcasing the assembly of the locking system when integrated in the series elastic differential. Locking the series elastic differential involves pushing the locking pins down to block the output attachments from rotating. This is illustrated in **(F)**.

The whiffletree used with the locking mechanism consists of three bars: one bar connects the index and middle fingers (bar 1), one bar connects the ring and pinky fingers (bar 2), and the main bar (bar 3) connects bar 1 and bar 2, as depicted in Figures 7A,B. In this mechanism, the adapted whiffletree upon contact of one finger with the environment or the object surface, the whiffletree facilitates the motion of the rest unconstrained fingers. The whiffletree allows one motor to control multiple fingers in a coordinated fashion, so a small linear displacement of the tendon causes appropriate proportional angular displacements at all robot joints. The whiffletree has been appropriately designed with protruding pins on the top two bars of the whiffletree that interact with the elongated buttons. When pressed, the button restricts the motion of the whiffletree by blocking the pins from moving. Similar to the whiffletree locking mechanism, the buttons were employed to block the rotational motion at the outputs of the four-output gear differential and the series elastic differential. Utilizing a similar principle to the whiffletree, the four-output gear differential and the series elastic differential can both be fitted with protruding pins. The pins allow the button locking mechanism to obstruct the differential outputs, facilitating the execution of multiple grasping postures and gestures. This locking mechanism was expanded and integrated into the four output gear differential and the series elastic differential, providing an improved means of controlling the differentials outputs.

A total of 16 different finger combinations can be implemented using the selectively lockable differential mechanism. A single motor, which is combined with the six discrete positions of the thumb, can produce a total of 96 different grasping postures and gestures.

#### 2.3.2. Automated Selectively Lockable Differential

Similarly to the manually selectively lockable differential, the automated selectively lockable differential utilizes an alternative mechanism capable of facilitating the execution of multiple selectable grasping strategies. Unlike the manually selectively lockable differential, the automated lockable differential uses a small, low torque micro-servo (DFRobot DF 9 g micro-servo) to select the desired differential outputs, rather than manually locking and unlocking buttons in place. The active locking allows the implementation of controllable whiffletrees to be fully automatic in prosthetic hands. This enables amputees to perform bimanual tasks with increased efficiency, as the opposite hand is not required to adjust the grasp pose of the prosthetic hand before the task, since the pose can be selected autonomously during the task. To showcase automated locking, the selectively lockable whiffletree differential was used to select various finger combinations that can facilitate the execution of efficient grasps with underactuated prosthetic hands.

**Figure 8:**
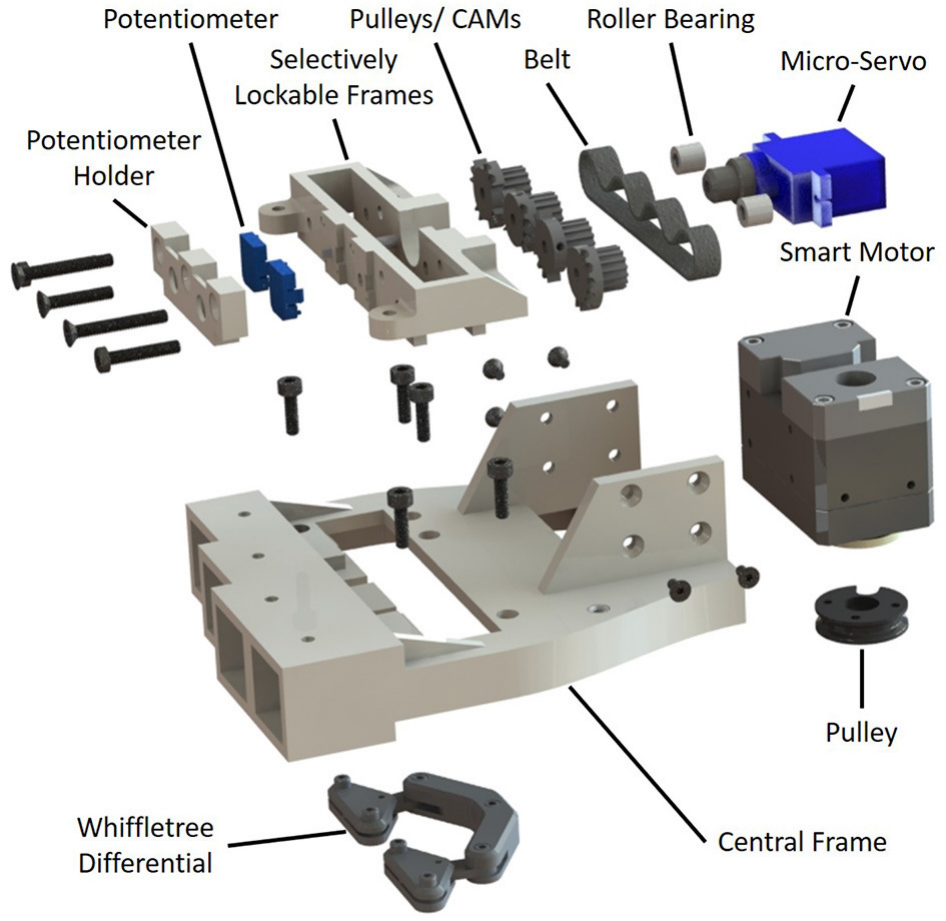
The automated selectively lockable differential consists of two main mechanisms: a selector mechanism that rotates so as to select the differential output behavior and a whiffletree differential for distributing the input load evenly across four outputs. The selector is composed of four pulley/cams, two roller bearings, two potentiometers, a belt, an input pulley, a micro-servo, and a selector frame.

**Figure 9:**
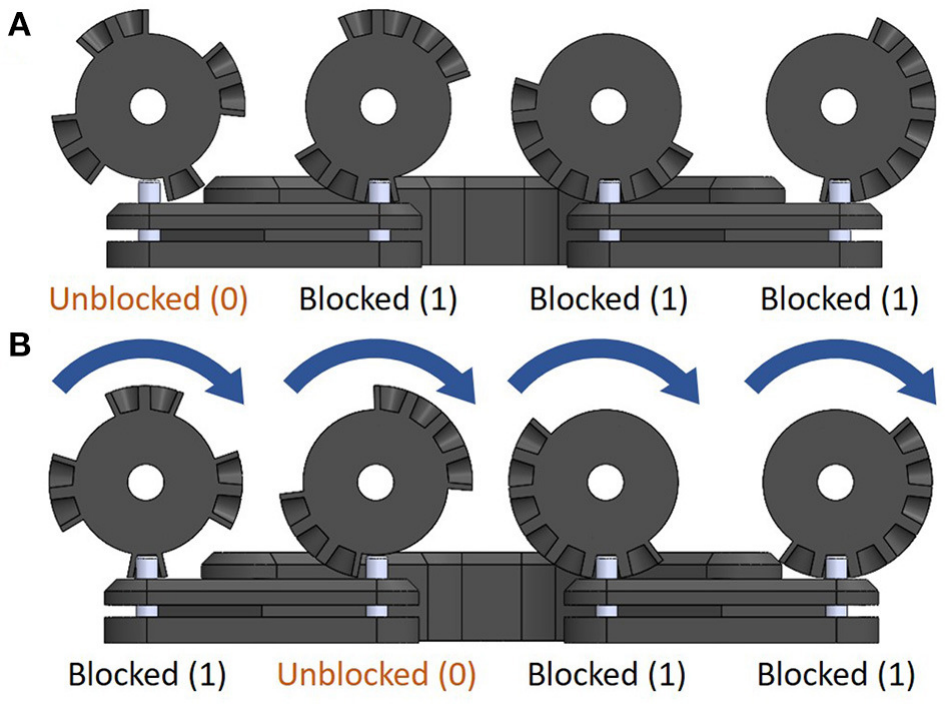
The automated selectively lockable differential performs controllable locking by blocking and unblocking the motion of the whiffletree. This is depicted in **(A)**. In **(B)** the cams are rotated in sync with the micro-servo so as to provide the desired differential output.

The locking mechanism is composed of four pulleys, a belt, a single actuator, two potentiometers, and a whiffletree differential as seen in Figure 8. The whiffletree differential output is connected to four fingers (index, middle, ring, and pinky), while the input of the differential is connected to a single Dynamixel XM430-W350-R smart motor. Each pulley contains a different cam profile, which rotates in sync while interacting with the whiffletree differential's protrusions, providing obstructed and unobstructed tendon motion at the whiffletree outputs. This can be seen in Figure 9. In order to organize these combinations effectively, such that the cam profile is strong and less prone to error during output selections, a gray code format is used over a binary code format. Two potentiometers are connected out of phase from each other on two cams to detect the lockable mechanism's current combination over a complete revolution. A total of 16 finger combinations can be achieved with the four fingers (index, middle, ring, and pinky). Although this system is implemented for a whiffletree differential, the locking mechanism can be adapted to accommodate other differential mechanisms.

## 3. Experiments and Results

Different experiments were conducted to assess the performance of the proposed differential mechanisms. The first experiment evaluated how much the fingertip force exertion capabilities were affected when the fingers where selectively locked. The second experiment focused on assessing the selectively lockable differential mechanisms capability in providing various hand gesture combinations with the different differentials. The third experiment assessed the grasping capabilities of the differentials when they are integrated into prosthetic hands. The fourth and fifth experiments focused on evaluating the maximum tendon tension and maximum tendon displacement that can be achieved at the outputs of the differentials.

**Figure 10:**
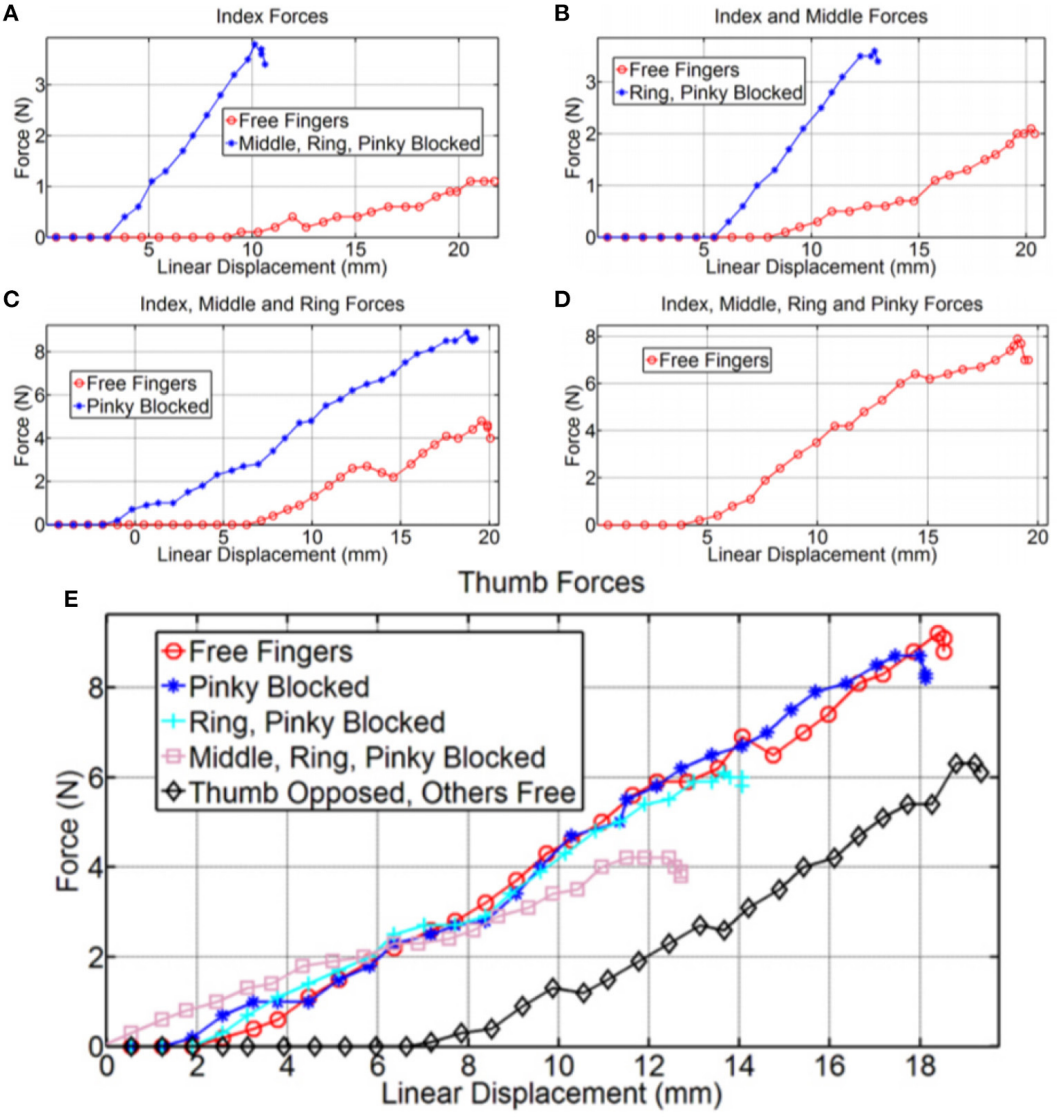
The relationship between tendon displacement and finger forces for different grasp poses are compared with blocked and unblocked fingers. **(A)** Shows the force output when only the index is unblocked. **(B)** Depicts the force exerted when only the middle finger is unblocked. Similarly, **(C)** Presents the forces but with only the pinky finger experiencing blocking. The force output when all fingers are unblocked can be seen in **(D)**. A comparison of all forces is illustrated in **(E)**.

The force exertion experiments were conducted on the selectively lockable differential to investigate the effect on force exertion when the differential mechanism experiences locking/blocking. The relationship between displacement at the input and force exertion at the outputs, is presented in Figure 10 with different finger combinations being compared. When blocking the fingers we are able to maximize the force applied by the free fingers at there fingertips (e.g., precision grasps). If needed the user can utilize this behavior to maximize the force transmitted from the servo motor to the fewer active fingertips.

### 3.1. Gesture Execution Experiments

**Figure 11:**
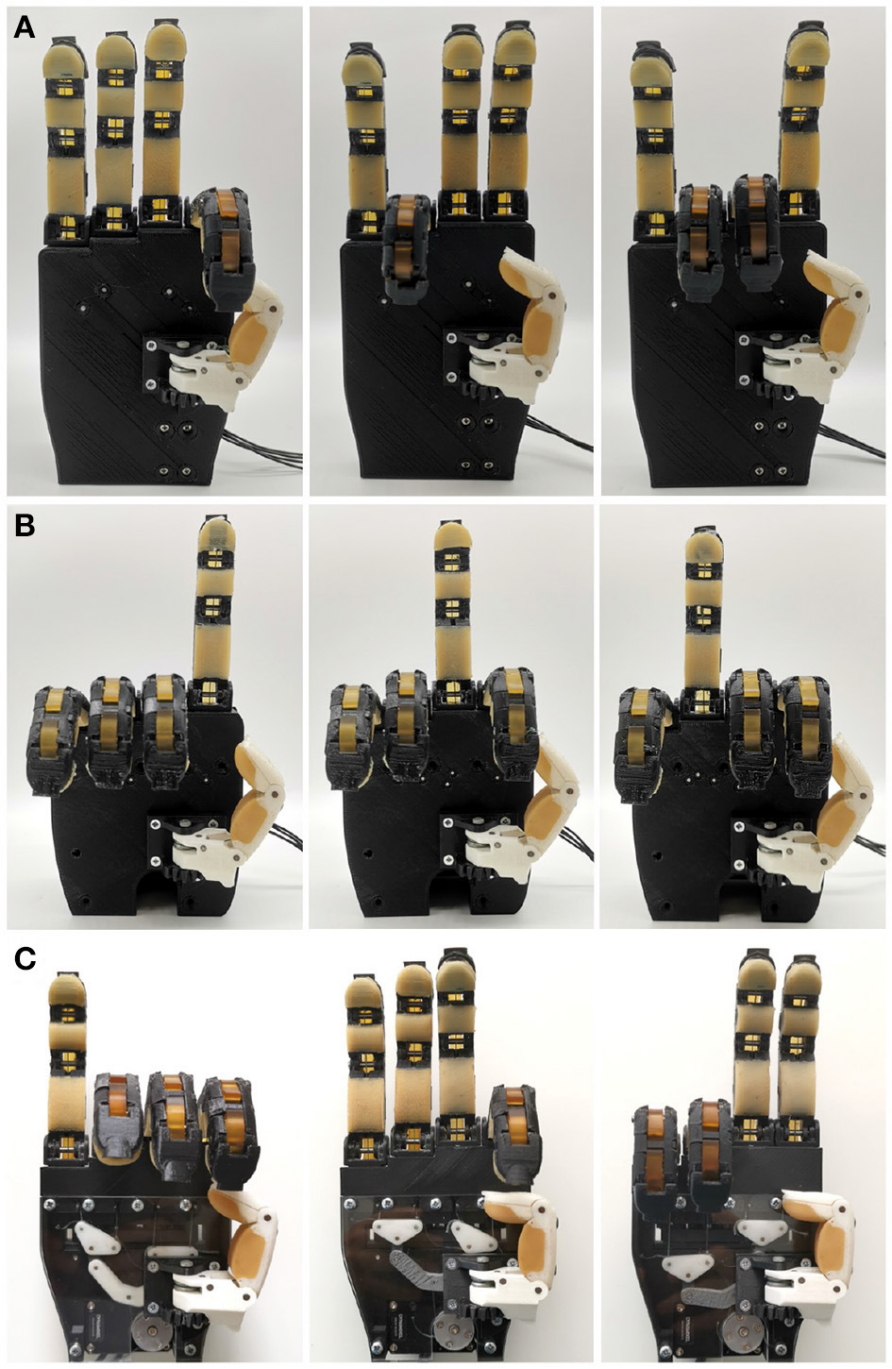
Hand gesture combinations executed by a prosthetic hand equipped with a selectively lockable differential mechanism. The Locking mechanism was implemented on the four output geared differential **(A)**, the series elastic differential **(B)**, and the whiffletree **(C)** on similar prosthetic hands, altering the index, middle, ring, and pinky fingers flexion combination patterns.

The second experiment assessed the proposed selectively lockable differentials capabilities in executing various grasp poses and hand gestures. To evaluate the abilities of the selectively lockable mechanisms to enhance the performance of all the proposed differentials, the mechanisms were incorporated into a prosthetic hand with a single actuator so as to demonstrate the different achievable hand poses. To showcase the different grasp postures, the buttons of the selectively lockable differentials were locked into different combinations. The three differentials were capable of achieving the full 16 different combinations. This is depicted in Figure 11. The importance of controlling the differential's outputs is critical for selecting grasping strategies and allows: i) different hand gestures to be signed, ii) reaching an object in a narrow space, or iii) executing non-prehensile manipulation tasks (e.g., pressing buttons or moving sliders).

### 3.2. Grasping Performance Experiments

**Figure 12:**
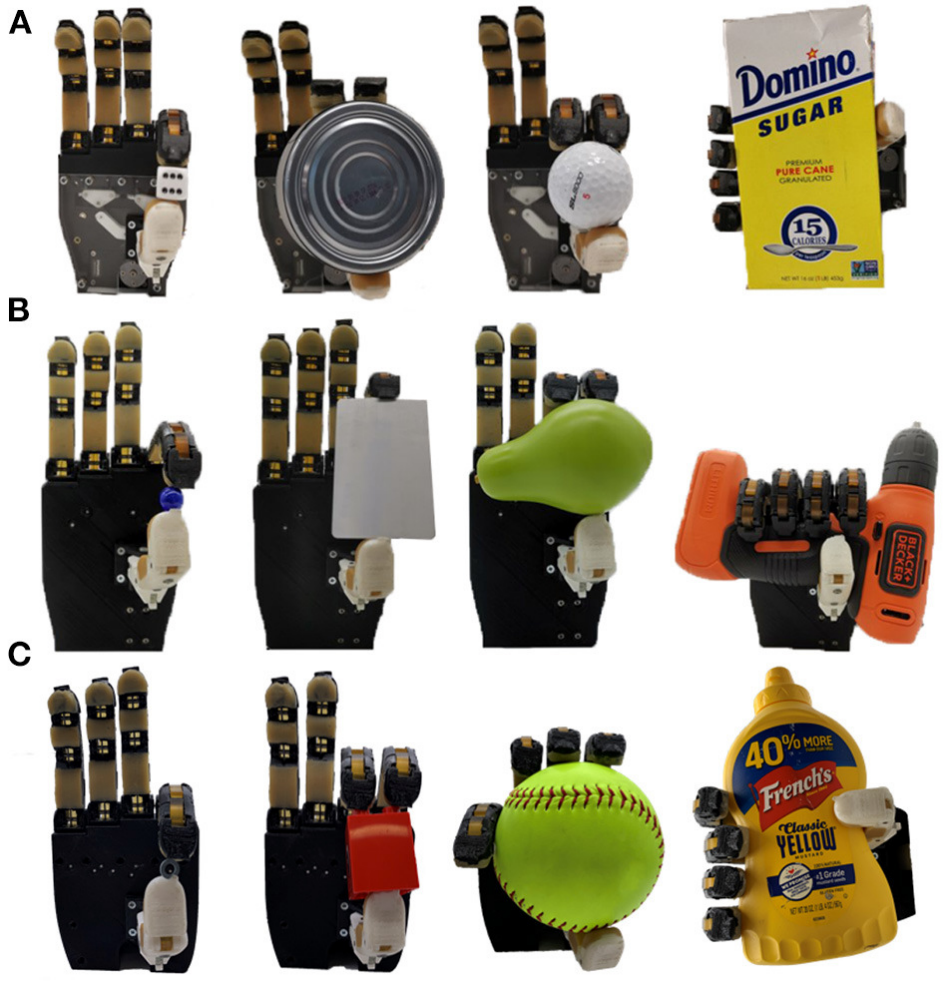
Grasping experiments conducted with the three prosthetic hands equipped with the proposed differential mechanisms whiffletree **(A)**, four output gear differential **(B)**, and series elastic **(C)**. The three differentials can be seen allowing a prosthetic hand to execute a variety of grasping strategies (pinch, tripod, and power grasps).

The third experiment was conducted to evaluate the ability of the differentials to improve the grasping performance of prosthetic hands in executing activities of daily living. To do so, the YCB object set designed by Calli et al. (2017), was used to evaluate the grasping efficiency of the prosthetic hands with the proposed differentials integrated. Twelve objects from the object set were selected: a credit card, a washer, a dice, a marble, a tuna fish can, a golf ball, a pear, a Lego Dublo block, a mustard bottle, a box of sugar, a drill, and a baseball. All hands were capable of grasping all twelve objects. This can be seen in Figure 12, where the selectively lockable differential mechanism allows the hand to execute different grasping postures, achieving optimal grasping performance for the encountered objects.

### 3.3. Tendon Tension Experiments

The fourth experiment focused on testing the mechanical limits of the designed differentials. The experiment consisted of measuring the tendon's tension until either the tendon, the differential, or the motor failed. Hanging weights of increasing masses were attached to the output ends of the differentials until it was unable to lift the weight. To perform the experiment, equal weights of 100 g were incrementally added at the end of the tendon in all four outputs while the differential was running until the system could not withstand the load. The results of the total exerted forces of all four outputs of each differential are provided in Table 1. The whiffletree differential used in the developed prosthetic hands in section 3.1, was capable of holding up to 42.8 N of tendon tension per output before failure. The four-output geared differential obtained a maximum tendon tension of 39 N per output during the experiments. Although the maximum theoretical tendon tension calculated in section 2.1 can be more than 100 N per output, the calculation does not consider efficiency loss due to friction between components, the operating conditions of the motor, or the mechanical resistance of the components used in the differential. When using the series elastic differential it is capable of switching between a rigid and a compliant mode allowing the differential to select when the elastic elements should be used. The maximum tendon tension force of 53.8 N per output was achieved when in the rigid mode, while a maximum force of 45.8 N per output was obtained for the compliant mode. Note that the stiffness of the elastic element of the series elastic differential mechanism can be selected according to the requirements of the application.

**Table 1:** Comparison of the proposed differentials.

**Differentials**	**Whiffletree differential**	**Four-output differential**	**Series elastic differential**
Inputs	1	1	1
Outputs	4	4	4
Total displacement	Limited^a^	Continuous^b^	Continuous^b^
Displacement between outputs	Limited^c^	Continuous^b^	Limited^d^
Size^e^ (mm)	23 x (54 + T) × 81	41 × 100 × 41	57 × 23 × 23
Weight (g)	46	169.8	22.5
Max force output^f^ (N)	171	156	215 (rigid mode)

a *The total displacement of the whiffletree is limited by the translation length available*.

b *The rotary motion of the mechanism allows for continuous winding at the outputs*.

c *The adaptability of the differential is limited by the length of the whiffletree bars*.

d *The adaptability is limited by the max compression displacement of the elastic elements*.

e *T is the travel distance needed for the mechanism to adapt*.

f *The max force output is the total force of all outputs*.

### 3.4. Tendon Displacement Experiment

The last experiment focused on measuring the amount of achievable displacement in each output of the three differential mechanisms. This displacement is important as it offers the required adaptability needed for grasping a wide range of objects, conforming to the object shape, and maximizing the contact patches between the fingers and the object surface, increasing also grasping quality. The three differentials were actuated in an unblocked state to achieve the maximum obtainable displacement at the differentials outputs. Additionally, the three differentials were also tested with three of the four outputs being blocked, allowing for the minimum achievable tendon displacement to be measured. When unblocked the whiffletree was capable of 21 mm of tendon displacement, but was only limited by the available translation length, which is limited by the length of the palm of the prosthetic hand. In the second test scenario where three of the four outputs are blocked, the whiffletree differential was able to obtain a displacement of 10 mm, which was limited by the length of the upper whiffletree bars. The four-output geared differential was able to perform continuous rotations at the outputs in both locked and unlocked scenarios providing continuous displacement. The maximum tendon displacement of this design is only limited by the amount of tendon the pulleys at the output shafts can hold. The series elastic differential when unblocked is capable of continuous rotation similarly to the four-output geared differential, but this continuous rotation only applies to cases when all four outputs are allowed to move continuously. When one or more outputs is blocked, the series elastic differential can only provide displacements up to 43 mm.

## 4. Discussion

Two differential mechanisms and four different locking mechanisms have been proposed, each capable of improving the grasping capabilities of prosthetic hands in different circumstances. The selectively lockable differentials offer increased controllability of the differential outputs facilitating the execution of all 16 finger flexion/extension combinations (e.g., controlled flexion across the index, middle, ring, and pinky fingers on a prosthetic hand). For grasps, which do not need the involvement of all four fingers (index, middle, ring, and pinky) to oppose the thumb to complete the grasp, the subsidiary fingers can be blocked to maximize the force transmitted to the active fingers by the motor. The developed selectively lockable differentials have been designed to accommodate different user requirements. The manual selectively lockable differential utilizes manually lockable buttons meaning the design does not require additional electronics and actuators to use the mechanism. Hence, utilizing a body-powered approach enables the mechanism to significantly reduce the cost of implementation in a prosthetic device where the price is an essential element. Although the automated selectively lockable differential requires an additional actuator to operate, unlike the manually lockable whiffletree differential, this actuator does not need a high torque rating as the high loads exerted by the differential are parallel to the axis of the actuator. This allows the chosen actuator to be small and compact, reducing the size, and cost of the total system significantly. The increased autonomy offered by the system's active approach allows the use of selectively lockable differential mechanisms to increase efficiency in bi-manual tasks for amputees and reduce intervention and effort needed to switch the gesture or grasp pose of the hand. The automated selectively lockable differential can also be adopted in robotic systems, where full autonomy is required.

Other than the whiffletree differentials, which have a limited range of motion, rotary mechanisms like the four-output gear differential grant continuous rotation at the outputs. The benefit of using a rotary mechanism is its ability to operate within a fixed volume size. In contrast, traditional pulley and whiffletree differential mechanisms require additional space to accommodate the mechanism's translational motion. This is generally not an issue in anthropomorphic prosthetic hand designs (Laliberté et al., 2002; Weiner et al., 2018), where a large plane usually is available to accommodate the movements of the pulley and whiffletree differentials. However, for prosthetic devices that require large displacements at the differential outputs to reach their maximum range of motion, pulley and whiffletree differentials are not sufficiently compact.

Finally, the series elastic differential offers a simpler and smaller solution than the four-output differential via the implementation and utilization of passive elastic elements. This results in a mechanism with fewer components and reduced weight. However, passive elastic elements in series with the actuator output can produce a parasitic force reducing the maximum achievable force output. This is because the actuator must use some energy to compress the elastic element before achieving the desired differential displacement. To overcome this, the series elastic differential has been developed such that the differential is capable of switching between a compliant and adaptive mode and a rigid mode based on the rotating direction of the connected actuator. The ability to switch between compliant and rigid modes led to a force output difference of up to 17.4%. Similar to the whiffletree differential, where the maximum displacement between outputs is limited by the bar length, for the series elastic differential, this is constrained by the circumference of the main body and the maximum compressible length of the elastic elements. Thus, this design choice limits the differential's maximum adaptability. However, the total displacement of the series elastic differential is continuous if all outputs wind together. In contrast the whiffletree differential also has a limited total displacement, which is constrained by the operating volume allocated for the differential to translate in. The four-output gear differential, is capable of independently rotating each output continuously until all four outputs experience an equal load, where it will then wind the outputs together providing a continuous total displacement. A comparison of the proposed differential systems is presented in Table 1.

## 5. Conclusion

In this paper, we presented a set of lightweight and compact differential mechanisms for prosthetic hands where low weight, small size, and affordability are key requirements for a successful design. Locking mechanisms for improving the controllability of the three examined differentials (a four-output geared differential, a series elastic differential, and a whiffletree differential) were developed. Two different locking approaches were implemented with one being manual and the other using a small low torque actuator to allow for active control. The locking mechanisms facilitated all 16 different finger flexion and extension combinations (across the index, middle, ring, and pinky fingers). The four-output geared differential was developed in a compact manner allowing for the development of lightweight prosthetic hands. The proposed device is capable of exerting 39 N of tendon tension per output. The final differential type developed is a series elastic differential that is capable of switching between a compliance mode for adaptive behavior and a power mode for a non-adaptive behavior which is capable of exerting up to 17.5% more force. The tendon tension per output of the differential was 45.75 N in its compliance mode and 53.75 N in its power mode. All differentials are experimentally tested and compared.

Regarding future directions, we plan to integrate the automated locking into more differentials such as the four-output gear differential and the series elastic differential. Our future work will also focus on equipping the fingers with appropriate tactile and force torque sensors as well as on further evaluating how underactuation affects grasping quality and grasp stability through a forces-oriented quantitative analysis. Such an analysis will require redesigning all the utilized prostheses to accommodate the sensing elements and a series of new experiments and comparisons. Finally, we also intend to integrate the proposed differential mechanisms in devices other than prosthetic hands in order to showcase all feasible use cases.

## Data Availability Statement

Publicly available datasets were analyzed in this study. This data can be found here: https://github.com/newdexterity/Differentials.

## Author Contributions

GG contributed to the design of the selectively lockable differential mechanisms. MS developed the series elastic differential. LG developed four-output geared differential. GK contributed to the development of the manually selectively lockable whiffletree differential. ML contributed on the ideas and supervised. GG, MS, LG, and GK in the implementation of the different differentials. The manuscript were prepared by the authors collectively. All authors contributed to the article and approved the submitted version.

## Conflict of Interest

The authors declare that the research was conducted in the absence of any commercial or financial relationships that could be construed as a potential conflict of interest.

## Publisher's Note

All claims expressed in this article are solely those of the authors and do not necessarily represent those of their affiliated organizations, or those of the publisher, the editors and the reviewers. Any product that may be evaluated in this article, or claim that may be made by its manufacturer, is not guaranteed or endorsed by the publisher.
